# Genetic Variants as Predictors of the Success of Colorectal Cancer Treatments

**DOI:** 10.3390/cancers15194688

**Published:** 2023-09-22

**Authors:** Koldo Garcia-Etxebarria, Ane Etxart, Maialen Barrero, Beatriz Nafria, Nerea Miren Segues Merino, Irati Romero-Garmendia, Ajay Goel, Andre Franke, Mauro D’Amato, Luis Bujanda

**Affiliations:** 1 Biodonostia, Gastrointestinal Genetics Group, 20014 San Sebastián, Spain; 2 Centro de Investigación Biomédica en Red de Enfermedades Hepáticas y Digestivas (CIBERehd), 08036 Barcelona, Spain; luis.bujandafernandezdepierola@osakidetza.eus; 3 Biodonostia, Gastrointestinal Disease Group, Universidad del País Vasco (UPV/EHU), 20014 San Sebastián, Spain; ane.etxartlopetegi@osakidetza.eus (A.E.); mbarrero@onkologikoa.org (M.B.); beatriz.nafriajimenez@osakidetza.eus (B.N.); nereamiren.seguesmerino@osakidetza.eus (N.M.S.M.); 4 Department of Genetics, Physical Anthropology and Animal Physiology, University of the Basque Country (Universidad del País Vasco/Euskal Herriko Unibertsitatea), 48940 Leioa, Spain; 5 Department of Molecular Diagnostics and Experimental Therapeutics, Beckman Research Institute, City of Hope Comprehensive Cancer Center, Duarte, CA 91010, USA; ajgoel@coh.org; 6 Institute of Clinical Molecular Biology, Christian-Albrechts-University of Kiel, 24105 Kiel, Germany; a.franke@ikmb.uni-kiel.de; 7 Gastrointestinal Genetics Lab, CIC bioGUNE, Basque Research and Technology Alliance, 48160 Derio, Spain; mdamato@cicbiogune.es; 8 IKERBASQUE, Basque Foundation for Sciences, 48009 Bilbao, Spain; 9 Department of Medicine and Surgery, LUM University, 70010 Casamassima, Italy

**Keywords:** colorectal cancer, outcomes, genetics, markers, survival, treatment, genetic association

## Abstract

**Simple Summary:**

Some colorectal cancer (CRC) outcomes are partially associated with genetics, and different studies have proposed several genetic variants as predictors. However, analysis of their performance in other populations is limited. Thus, our objectives were to assess their use in our cohort and to find additional genetic variants associated with CRC outcomes. We found that some of the genetic variants proposed as predictors could be used in our cohort, although the addition of clinical data improved the performance. In addition, we found additional genetic variants that could be useful to predict the CRC manifestations in our population. Our findings will help to refine the use of genetic polymorphisms to predict CRC outcomes in our population, and we expect that our findings could be useful for other populations.

**Abstract:**

Background: Some genetic polymorphisms (SNPs) have been proposed as predictors for different colorectal cancer (CRC) outcomes. This work aims to assess their performance in our cohort and find new SNPs associated with them. Methods: A total of 833 CRC cases were analyzed for seven outcomes, including the use of chemotherapy, and stratified by tumor location and stage. The performance of 63 SNPs was assessed using a generalized linear model and area under the receiver operating characteristic curve, and local SNPs were detected using logistic regressions. Results: In total 26 of the SNPs showed an AUC > 0.6 and a significant association (*p* < 0.05) with one or more outcomes. However, clinical variables outperformed some of them, and the combination of genetic and clinical data showed better performance. In addition, 49 suggestive (*p* < 5 × 10^−6^) SNPs associated with one or more CRC outcomes were detected, and those SNPs were located at or near genes involved in biological mechanisms associated with CRC. Conclusions: Some SNPs with clinical data can be used in our population as predictors of some CRC outcomes, and the local SNPs detected in our study could be feasible markers that need further validation as predictors.

## 1. Introduction

Colorectal cancer (CRC) is the second most diagnosed cancer and the second cause of death among cancers, accounting for 10% of diagnosed cancers in developed countries [1]. Its risk is influenced by the environment, genetics, and microbial composition and can be sporadic or result from inflammatory processes [2,3,4]. Therefore, CRC is a significant public health issue, and strategies must be developed to predict the prognosis and adjust the treatment [5,6].

Different treatments are used in CRC, such as surgery or the use of chemotherapy. In the last few years, various drugs have been developed (e.g., 5-fluorouracil or capecitabine) that can be used alone or in combination to treat CRC. Among the different factors that could determine the success of those treatments, it has been observed that some genetic polymorphisms (SNPs) can affect success. SNPs of several candidate genes related to the biological mechanisms of the treatment have been analyzed to test their role in the success of the treatments: survival in FOLFIRI-based treatment [7], survival in Bevacizumab-based treatment [8], and toxicity to 5-fluorouracil and capecitabine [9,10,11,12,13,14,15,16,17,18,19]. In addition, genome-wide association analyses have been used to find SNPs associated with metastatic CRC survival in treatment with chemotherapy plus biologics [20], survival in rectal cancer [21], progression-free survival in metastatic CRC in different treatments [22], and survival in CRC [23]. However, there are discrepancies between studies, possibly due to the differences in the frequency of the risk variants between populations [6]. It has been proposed that SNPs related to toxicity could be associated with the efficacy of the treatment [24].

Previously, we analyzed CRC patients from a Basque cohort to study the performance of the available genetic information to assess the risk of developing CRC. In that study, we showed that the available genetic information could be used. Still, there were local genetic variants that could be relevant to the genetic architecture of CRC in our cohort [25].

Thus, our aim with this study is to assess if the polymorphisms previously associated with the success of the treatment in CRC are valid predictors in our cohort and to explore possible local genetic variants that could be predictors of the success of the treatment.

## 2. Materials and Methods

### 2.1. Recruitment

CRC cases were diagnosed using standard criteria, and the samples used in this study were obtained in standard clinical practice after signing an informed consent letter at Hospital Universitario Donostia (San Sebastian, Spain). In total, 869 cases were recruited. The present study was approved by the Local Ethics Committee (Comité de Ética de la Investigación con medicamentos de Euskadi, code: PI+CES-BIOEF 2017-10).

### 2.2. Genotyping

The genotyping of the DNA samples analyzed in this work was carried out using the Illumina Global Screening Array through the Illumina iScan ((llumina, San Diego, CA, USA) high-throughput screening system at the Institute of Clinical Molecular Biology (Kiel, Germany). Illumina GenomeStudio software (v2.0) and its GenCall algorithm were used to transform raw intensities into alleles.

Quality control of the called genotypes and samples was carried out using the following filters: The exclusion of samples with ≥5% missing rates; markers with non-called alleles; markers with missing call rates > 0.05; related samples (PI-HAT > 0.1875); samples whose genotyped sex could not be determined; and samples with a high heterozygosity rate (more than three times the SD from the mean). In addition, autosomal SNPs were kept, and markers with Hardy-Weinberg equilibrium *p* < 1 × 10^−5^ were removed. Finally, principal component analysis was used to identify outlier samples (deviation of more than six times the interquartile range) through FlashPCA (v2.0) [26].

The Sanger Imputation Service was used to impute additional SNPs. For that, release 1.1 of the Haplotype Reference Consortium was used as a reference panel, and the EA-GLE2+PBWT pipeline was used to carry out the imputation [27,28,29]. The imputed variants were filtered using the following criteria: variants with an INFO score < 0.80, a MAF score < 0.01, and non-biallelic markers were removed.

After the QC of the imputed data, 5,399,981 SNPs from 833 cases were kept.

### 2.3. Analyses

We analyzed seven outcomes (1-year survival, 3-year survival, 5-year survival, 5-years without relapse, 5-years without relapse in patients treated with 5-fluorouracil-based chemotherapy, 5-years without relapse in patients treated with capecitabine, and 5-years without relapse in patients without chemotherapy) and the use of chemotherapy. In addition, we analyzed separately the stage of CRC (I+II and III+IV) and location (right colon, left colon, and rectum) for the same treatments and outcomes.

For each analysis, we analyzed the performance of 63 SNPs previously associated with CRC outcomes (Supplementary Table S1). We retrieved those SNPs from the GWAS Catalog [30], specifically from the studies GCST011584 [20], GCST002820, GCST002821 [21], GCST003057, GCST003058 [22], and GCST003229, GCST003230, and GCST003231 [23]. In addition, SNPs associated with survival in FOLFIRI-based treatment [7], survival in Bevacizumab-based treatment [8], and toxicity to 5-fluorouracil and capecitabine [9,10,11,12,13,14,15,16,17,18,19] were analyzed. We used a generalized linear model to test if the carriership of the tested allele affected a given outcome. We used the area under the curve (AUC) of the receiver operating characteristic curve to measure the performance. Three AUCs were calculated: using only the carriership of the tested allele as a predictor; using sex, age, and tumor stage as predictors; and using the carriership of the tested allele, sex, age, tumor stage, and the first four principal components of the genetic distance of individuals as predictors. Those analyses were carried out using the R language [31] and the package pROC [32].

Moreover, for each outcome, a genome-wide association analysis was performed using logistic regression implemented in Plink [33], adjusting by sex, age, and the first four principal components of the genetic distance of individuals, stage, and location. In the case of the analyses of outcomes by stage, the analyses were adjusted by sex, age, location, and the first four principal components of the genetic distance of individuals; and in the case of the analyses of outcomes by location, sex, age, stage, and the first four principal components of the genetic distance of individuals.

## 3. Results

The demographic and clinical characteristics of each outcome we have analyzed are shown in Table 1. On the whole, there were significant differences in age, stage, location, lymph, and metastasis in each outcome but not in sex or histologic grade (Table 1).

### 3.1. Performance of Genetic Variants Previously Associated with CRC Outcomes

From the 63 SNPs previously associated with various CRC outcomes (Supplementary Table S1), 26 of them showed an AUC > 0.6 and a significant association (*p* < 0.05) with one or more outcomes analyzed in the present work (Table 2). In addition, the AUC of the SNPs was improved with the inclusion of additional variables (sex, age, and genetic distance).

The most significant SNP was rs13180087 T > C (Table 2), whose minor allele was more prevalent in patients with left colon tumors without chemotherapy and who relapsed after 5 years (OR = 17, *p* = 3 × 10^−4^). It was followed by rs17057166 C > T, whose minor allele was more prevalent in patients with I+II stage tumors and did not survive 1 year (OR = 4.3, *p* = 8 × 10^−4^) (Table 2).

Regarding the best performance, rs1555895 A > G had an AUC of 0.7 to differentiate patients with rectal cancer that could have no 5-year relapse when they have not been treated with chemotherapy (Table 2). However, the AUC calculated with clinal variables (sex, age, and stage) outperformed the AUC using only the genetic variant (AUC = 0.76). In fact, in the majority of the cases, the clinical variables were more informative than only the SNP, except for rs11246159 T > C in the 5-year relapse of patients with I+II tumors and rs885036 A > G in the 5-year relapse of patients with left colon tumors treated with capecitabine (Table 2). In addition, when genetic data and clinical data are combined, the AUC outperformed the AUC values separately, reaching high values such as rs17048372 G > T and rs1801265 A > G in the 5-year relapse of patients with right colon tumors treated with 5-fluorouracil (AUC = 1) (Table 2).

Moreover, some SNPs were associated with outcomes other than those previously associated with them (Table 2). For example, rs17057166 C > T or rs3781663 G > A were associated with survival in rectal cancer, and our cohort was associated with survival in right or left cancer. The SNP rs1128503 A > G, which is associated with the toxicity of capecitabine, was associated with the success of using 5-fluorouracil. In addition, the effect of some genetic variants was not the same as in the study they were described (e.g., rs1573948 T > C, rs3781663 G > A, or rs1555895 A > G), or depending on the outcome, the effect was different (e.g., rs11246159 T > C, rs885036 A > G, or rs7299460 C > T).

### 3.2. Discovery of Local Genetic Variants Associated with CRC Outcomes

Apart from analyzing the performance of SNPs described in the literature, we searched for SNPs associated with CRC outcomes in our cohort. We did not find any genome-wide significant (*p* < 5 × 10^−8^) SNPs, and we found 49 suggestive (*p* < 5 × 10^−6^) loci associated with one or more CRC outcomes (Table 3).

The most significant SNP was rs10845123 G > A, associated with 5-year survival (OR = 2.9, *p* = 9.6 × 10^−9^) and located in the *KLRK1-AS1* gene. The next more significant SNPs were rs6889868 T > C, which was associated with 3-year survival (OR = 3.2, *p* = 6.4 × 10^−7^) and located in the intergenic region; rs61991339 T > C, which was associated with 1-year survival (OR = 4.2, *p* = 7.7 × 10^−7^) and located in the *UNC79* gene; and rs6088387 G > T, which was associated with the use of chemotherapy (OR = 6.5, *p* = 7.9 × 10^−7^) and located in the *RALY* gene.

Moreover, some SNPs were associated with the outcome due to the effect of one subgroup (Table 3). For example, the association of rs1347485 A > G with 3-year survival (OR = 8.3, *p* = 1.6 × 10^−6^) was driven by its association in patients with I+II stage tumors (OR = 16.8, *p* = 1.9 × 10^−6^); the association of rs4712605 A > G with 1-year survival (OR = 4.9, *p* = 2.9 × 10^−6^) was driven by its association in patients with I+II stage tumors (OR = 11.9, *p* = 4.1 × 10^−6^); and the association of rs9788099 G > A with 3-year survival (OR = 3.3, *p* = 3.1 × 10^−6^) was driven by its association in patients with rectal cancer (OR = 9.1, *p* = 2.6 × 10^−6^).

Finally, the suggestive SNPs associated with various CRC outcomes were located in introns of genes, upstream or downstream of genes, or intergenic regions (Table 3). However, rs17821546 A > G, associated with 3-year survival in patients with I+II stage tumors (OR = 21.9, *p* = 2.2 × 10^−6^), is located in the 3′UTR region of the *SULT1C2* gene.

## 4. Discussion

In this study, we have analyzed the performance as predictors of known SNPs associated with CRC outcomes in our cohort, as well as searched for new SNPs that could be used as predictors of CRC outcomes in our cohort.

We are aware that the sample size, especially for some outcomes, was limited. This limitation means that the most relevant effects (e.g., high ORs) are detected as significant or that sampling biases may be generated. Therefore, in the analyses of previously known genetic markers, the *p*-values should be interpreted in that context. In addition, significant signals at the genome-wide level (*p* < 5 × 10^−8^) are not found, probably due to the sample size, although suggestive signals (*p* < 5 × 10^−6^) could be detected. Therefore, the present study’s results should be validated, and follow-up analyses are needed in a larger cohort. However, considering our previous findings on the risk of CRC in this cohort [25] and the possible use of genetic variants to tailor treatments [6], we thought that this study could be a first step for our population to find appropriate genetic markers to predict CRC outcomes. In addition, in our previous studies of our population [25,34], we have detected local genetic variants that could be informative but that could not be detected in broader cohorts. Moreover, we are aware that the genetic particularities of our population due to its evolutionary history affect the generalization of the results obtained in this work. The isolation and the genetic drift have caused the frequencies of the alleles of the Basque population to be more similar to populations that lived in Europe in the Neolithic [35] or Iron Age [36] than modern European populations, whichd were impacted by migrations associated with Steppe pastoralism. Therefore, the SNPs that could be useful in our population could not be relevant for other populations, as it has been proposed previously to explain the differences in the results between populations [6]. Although a limitation, this observation could highlight the importance of analyzing local populations to assess the utility of known genetic markers and to find local genetic markers.

The genetic variants previously associated with CRC outcomes have variable performance. Some of them had a good performance and, therefore, can be used to predict some outcomes. In addition, some SNPs helped predict different outcomes. It has to be highlighted that the performance would improve if additional variables were included. Thus, more genetic information is needed to make a good prediction, and other clinical data must be used for a robust prediction.

Moreover, we have detected genetic variants that could be useful in predicting CRC outcomes in our cohort. The most significant signal was detected in an SNP (rs10845123) associated with 5-year survival and located in the *KLRK1-AS1* lncRNA. This lncRNA encodes a polypeptide regulated by TP53, which is involved in cell proliferation through its regulation of the cell cycle in DNA damage response [37]. Another significant signal was the SNP rs6088387, whose minor allele is associated with the risk of being treated with chemotherapy. This SNP is located in the *RALY* gene, a gene associated with CRC aggressiveness, and its expression is associated with a poor prognosis in CRC [38].

Other SNPs related to various outcomes were located in genes previously associated with CRC. For example, it has been detected that there is a higher expression of *GBP3* in CRC, although it is not a good predictor of response to immune checkpoint blockade [39]. The expression of *TSPAN11* has been associated with a stemness score and a stromal score of tumors in CRC [40], and it has been included in a model for prognosis prediction in CRC through its role in cell invasion [41]. In the case of *PTPRM*, it has been suggested that it may play a role in colorectal tumorigenesis since it regulates cell growth, and its loss promotes the growth of oncogenic cells [42]. In the case of other genes, their role in other cancers has been proposed. For example, the overexpression of *SULT1C2* has been associated with the growth, survival, migration, and invasiveness of hepatocellular carcinoma cells [43]. The expression of *PUS1* is associated with overall survival in hepatocellular carcinoma [44], and it has been described that *RBFOX3* plays a role in the chemosensitivity to 5-Fluorouracil in hepatocellular carcinoma [45].

On the whole, these results suggest that the genetic variants detected in our cohort could be feasible candidates to assist in the prediction of the outcome since the genes where they are located are associated with various biological mechanisms of CRC or other cancers.

It has to be pointed out that some of the SNPs detected, both in the analysis of SNPs previously associated with CRC outcomes and in the analysis of local genetic variants, were significant only in a specific stage or location (e.g., rs13180087 T > C or rs17057166 C > T), or the analyses of all patients altogether were driven by a specific stage or location (e.g., rs1347485 A > G or rs4712605 A > G). In the case of the risk of CRC, it has been observed that the genetic background is different depending on the location [25,46]. Thus, the use of those SNPs should consider the stage and location of the tumor to make an accurate prediction about a given outcome.

## 5. Conclusions

In conclusion, we have found that 26 genetic markers previously associated with CRC outcomes could be good predictors in our population and that the accuracy of the precision was improved using clinical data. In addition, we detected 49 local genetic variants that could be feasible markers for several CRC outcomes; however, considering our limited sample size, further validation is needed to assess their utility as predictors.

## Figures and Tables

**Table 1:** Demographics and clinical characteristics of the analyzed samples SD, standard deviation. size of the tumor in cm. grade and histologic grade of the tumor.

Outcome	1-Year Survival		3-Year Survival		5-Year Survival		5-Year Relapse		Chemotherapy		5-Fluorouracil—5-Year Relapse		Capecitabine—5-Year Relapse		No Chemotherapy—5-Year Relapse	
	No	Yes	No	Yes	No	Yes	Yes	No	Yes	No	Yes	No	Yes	No	Yes	No
N	102	731	183	650	241	592	63	509	398	431	91	127	125	201	130	293
Age (±SD)	78.2 (±11.4)	72.9 (±11.2)	76.9 (±11.3)	72.6 (±11.2)	77 (±11.1)	72.1 (±11.2)	71.5 (±11.5)	72.3 (±11.2)	70.9 (10.7)	75.8 (11.4)	73.2 (±11.5)	69.8 (±11)	76.3 (±10)	73 (±10.8)	79.3 (±11.2)	74.4 (±11.2)
*p*	1.9 × 10^−5^		8.9 × 10^−6^		1.3 × 10^−8^		0.6044		3.2 × 10^−10^		0.0280		0.0049		5.4 × 10^−5^	
Sex																
Female	34	270	67	237	81	223	21	194	140	163	37	44	40	75	45	115
Male	68	461	116	413	160	369	42	315	258	268	54	83	85	126	85	178
*p*	0.479		0.9702		0.2698		0.4599		0.4298		0.3649		0.3290		0.3646	
Stage																
I+II	31	449	56	424	88	392	33	356	124	354	33	84	54	133	85	265
III+IV	67	261	121	207	145	183	28	140	272	55	55	40	64	61	36	17
*p*	2.3 × 10^−9^		1.7 × 10^−17^		1.6 × 10^−15^		4.5 × 10^−3^		2.7 × 10^−57^		1.3 × 10^−5^		6.8 × 10^−5^		1.1 × 10^−10^	
Location																
Right	31	139	54	116	64	106	9	94	52	117	16	25	33	35	45	72
Left	18	201	35	184	49	170	12	149	86	131	23	37	20	65	28	99
Rectal	27	208	54	181	77	158	20	136	169	65	30	33	45	55	26	38
*p*	0.0103		0.0012		0.0032		0.2524		3.3 × 10^−18^		0.5232		0.0018		0.0061	
Size																
Size (±SD)	4.5 (±1.9)	3.8 (±1.9)	4.3 (±1.9)	3.8 (±1.9)	4.2 (±1.9)	3.8 (±1.9)	3.9 (±2.1)	3.8 (±1.9)	3.8 (±1.9)	3.9 (±1.9)	3.9 (±2)	3.6 (±1.7)	3.9 (±1.9)	3.6 (±2.1)	4.3 (±2.2)	3.8 (±1.9)
*p*	0.002		0.0036		0.0141		0.4992		0.2668		0.1442		0.2095		0.0372	
Grade																
Well	26	274	54	246	75	225	26	193	119	181	40	55	44	73	44	134
Moderate	74	469	126	417	161	382	42	328	279	261	58	86	86	128	90	166
Undifferentiated	12	86	26	72	30	68	10	55	56	41	17	25	15	16	11	28
*p*	0.1028		0.1087		0.3077		0.6514		0.0006		0.9593		0.5516		0.0639	
Lymph																
No	33	486	72	447	108	411	34	368	162	354	37	86	65	141	87	263
Yes	69	245	111	203	133	181	29	141	236	77	54	41	60	60	43	30
*p*	2.7 × 10^−11^		4 × 10^−13^		3 × 10^−11^		0.0027		9.8 × 10^−35^		7.1 × 10^−5^		0.0009		9.7 × 10^−9^	
Metastasis																
No	86	708	159	635	214	580	63	509	366	425	82	127	116	201	124	293
Yes	16	23	24	15	27	12	0	0	32	6	9	0	9	0	6	0
*p*	1.9 × 10^−8^		9.8 × 10^−10^		1.3 × 10^−8^		NA		4.8 × 10^−6^		0.0003		0.0001		0.0002	

**Table 2:** Performance of SNPs previously associated with CRC outcomes. A1, tested allele; Carriers, % of the carriers of the tested allele that showed the outcome; non-carriers, % of the Non-carriers of the tested allele that showed the outcome; OR (95% CI), odds ratio, and 95% confidence interval Direction: if the direction of the effect is the same (Same) or different (Diff) than the study where the SNP was described, “-” for non-data; *p*, *p*-value of the generalized linear regression; AUC SNP, AUC, and 95% of the confidence interval of the carriership of the tested allele as predictors; AUC clinical, AUC, and 95% of the confidence interval of sex, age, and stage as predictors; AUC Full, AUC, and 95% of the confidence interval of the carriership of the tested allele, sex, age, stage, and the first four principal components of genetic distance of individuals as predictors. Only SNPs with significant values and an AUC > 0.6 are shown.

SNP	A1	Outcome	Carriers (%)	Non-Carriers (%)	OR (95% CI)	Direction	*p*-Value	AUC SNP (95% CI)	AUC Clinical (95% CI)	AUC Full (95% CI)
rs898838	T	III+IV 5-fluorouracil—5-year relapse	67.86	41.67	4.7 (1.4–17)	Same	0.0136	0.61 (0.51–0.72)	0.68 (0.56–0.8)	0.77 (0.66–0.88)
		Left 5-fluorouracil—5-year relapse	47.37	9.09	33 (2.3–3635)	Same	0.0414	0.64 (0.54–0.74)	0.81 (0.69–0.93)	0.93 (0.86–1)
rs11246159	C	I+II 5-year relapse	5.33	12.14	0.4 (0.2–0.9)	Diff	0.0452	0.6 (0.52–0.68)	0.51 (0.4–0.62)	0.66 (0.56–0.76)
		Left 5-fluorouracil—5-year relapse	51.85	28.12	4.9 (1.3–23)	Same	0.0284	0.62 (0.49–0.75)	0.75 (0.63–0.88)	0.82 (0.71–0.92)
		Rectal 5-year relapse	4.69	18.89	0.2 (0–0.7)	Diff	0.0199	0.65 (0.56–0.74)	0.7 (0.58–0.83)	0.8 (0.7–0.89)
rs11644916	A	Rectal 5-year survival	43.04	25.19	2.7 (1.4–5.6)	Same	0.0049	0.6 (0.53–0.67)	0.78 (0.71–0.84)	0.81 (0.75–0.87)
		Rectal 3-year survival	32.91	15.56	3.1 (1.5–6.9)	Same	0.0035	0.62 (0.54–0.7)	0.78 (0.7–0.85)	0.82 (0.75–0.88)
		Rectal 1-year survival	20.25	5.93	5.3 (2–15)	Same	0.001	0.67 (0.57–0.77)	0.74 (0.64–0.85)	0.8 (0.71–0.88)
rs17057166	T	I+II 1-year survival	17.65	4.27	4.3 (1.8–9.9)	Same	8 × 10^−4^	0.65 (0.55–0.74)	0.71 (0.62–0.81)	0.75 (0.64–0.86)
		Right 1-year survival	33.33	14.29	3.9 (1.2–13)	Same	0.0276	0.6 (0.5–0.69)	0.8 (0.73–0.88)	0.88 (0.82–0.94)
		Right No chemotherapy—5-year relapse	63.64	31.46	7.3 (2.1–29)	Same	0.0024	0.61 (0.53–0.69)	0.69 (0.58–0.79)	0.83 (0.74–0.91)
rs1573948	C	Rectal 5-fluorouracil—5-year relapse	21.43	51.22	0.1 (0–0.7)	Diff	0.03	0.61 (0.51–0.72)	0.82 (0.71–0.93)	0.87 (0.77–0.96)
		Rectal Capecitabine—5-year relapse	11.76	54.69	0.1 (0–0.3)	Diff	0.0039	0.64 (0.56–0.72)	0.68 (0.55–0.8)	0.86 (0.78–0.94)
rs3781663	G	Right 1-year survival	11.34	27.27	0.2 (0.1–0.6)	Diff	0.0032	0.63 (0.53–0.73)	0.8 (0.73–0.88)	0.89 (0.83–0.94)
		Left 3-year survival	10.74	23.6	0.3 (0.1–0.7)	Diff	0.0039	0.62 (0.53–0.71)	0.77 (0.68–0.85)	0.81 (0.74–0.89)
		Left 1-year survival	3.31	15.73	0.1 (0–0.4)	Diff	9 × 10^−4^	0.69 (0.59–0.8)	0.74 (0.62–0.86)	0.86 (0.8–0.93)
rs1555895	A	Right 1-year survival	12.04	26.19	0.3 (0.1–0.9)	Diff	0.0321	0.61 (0.5–0.71)	0.79 (0.7–0.88)	0.86 (0.79–0.93)
		Rectal No chemotherapy—5-year relapse	25	68.42	0.1 (0–0.4)	Diff	0.0034	0.7 (0.58–0.83)	0.76 (0.63–0.9)	0.9 (0.82–0.98)
rs10152207	A	Right Capecitabine—5-year relapse	21.43	55.77	0.1 (0–0.7)	Diff	0.0291	0.61 (0.52–0.71)	0.71 (0.59–0.84)	0.84 (0.74–0.93)
rs17048372	T	Right 5-fluorouracil—5-year relapse	58.33	26.09	1127 (10–22986991)	Same	0.0314	0.66 (0.49–0.82)	0.89 (0.78–0.99)	1 (1–1)
		Rectal No chemotherapy—5-year relapse	71.43	25	6 (1.2–38)	Same	0.0356	0.68 (0.56–0.8)	0.81 (0.7–0.92)	0.93 (0.86–0.99)
rs13180087	C	Left 5-year relapse	20	5.3	6.5 (1.3–30)	-	0.0158	0.63 (0.47–0.78)	0.69 (0.55–0.83)	0.82 (0.7–0.93)
		Left Capecitabine—5-year relapse	41.18	18.75	5.7 (1.4–267)	-	0.0184	0.6 (0.48–0.72)	0.68 (0.54–0.82)	0.74 (0.63–0.86)
		Left No chemotherapy—5-year relapse	52.63	16.16	17 (4–86)	-	2 × 10^−4^	0.64 (0.54–0.74)	0.71 (0.6–0.81)	0.78 (0.66–0.89)
rs4377367	C	Left No chemotherapy—5-year relapse	31.91	15.28	3 (1.1–8.4)	Same	0.029	0.62 (0.51–0.72)	0.71 (0.6–0.81)	0.77 (0.67–0.87)
rs2936519	A	Left 5-year relapse	15.79	4.35	4.2 (1.1–16)	Same	0.0314	0.66 (0.5–0.82)	0.7 (0.55–0.84)	0.83 (0.73–0.92)
rs885036	A	Right No chemotherapy—5-year relapse	44.87	21.21	3.6 (1.2–12)	Same	0.029	0.61 (0.52–0.69)	0.69 (0.58–0.79)	0.8 (0.72–0.89)
		Left Capecitabine—5-year relapse	13.21	42.86	0.2 (0–0.5)	Diff	0.0028	0.69 (0.56–0.81)	0.68 (0.54–0.82)	0.8 (0.68–0.92)
		Rectal No chemotherapy—5-year relapse	33.33	55	0.2 (0–0.7)	Diff	0.0214	0.6 (0.48–0.72)	0.75 (0.63–0.88)	0.85 (0.75–0.95)
rs12224794	A	III+IV 5-fluorouracil—5-year relapse	49.15	74.19	0.3 (0.1–0.9)	-	0.0443	0.62 (0.52–0.71)	0.7 (0.59–0.8)	0.74 (0.64–0.84)
		Rectal No chemotherapy—5-year relapse	30.56	56.52	0.2 (0–0.8)	-	0.0267	0.63 (0.5–0.75)	0.78 (0.65–0.91)	0.84 (0.73–0.95)
rs1372474	G	Left 5-year relapse	23.08	5.76	10 (1.7–66)	Same	0.0094	0.6 (0.46–0.74)	0.69 (0.55–0.83)	0.83 (0.71–0.94)
rs1442089	C	Left 5-year relapse	23.08	5.8	10 (1.7–66)	Same	0.0094	0.6 (0.46–0.74)	0.69 (0.55–0.83)	0.83 (0.71–0.94)
rs1054190	T	Right Capecitabine—5-year relapse	30	56.52	0.2 (0–0.9)	Same	0.0402	0.61 (0.5–0.72)	0.71 (0.59–0.84)	0.82 (0.72–0.92)
rs7299460	T	III+IV No chemotherapy—5-year relapse	52	82.14	0.2 (0–0.8)	Same	0.0413	0.67 (0.54–0.81)	0.71 (0.56–0.85)	0.81 (0.67–0.94)
		Rectal Capecitabine—5-year relapse	55.77	31.82	2.7 (1–7.7)	Diff	0.0464	0.62 (0.52–0.72)	0.7 (0.59–0.81)	0.77 (0.68–0.87)
		Rectal No chemotherapy—5-year relapse	56.25	23.33	4.9 (1.3–22)	Diff	0.0248	0.67 (0.55–0.79)	0.75 (0.63–0.88)	0.86 (0.77–0.95)
rs3795897	A	I+II 5-fluorouracil—5-year relapse	50	26.03	3.6 (1.3–10.5)	Same	0.0173	0.6 (0.5–0.7)	0.64 (0.52–0.76)	0.72 (0.61–0.83)
		Left Capecitabine—5-year relapse	47.37	16.13	6.3 (1.6–27)	Same	0.01	0.66 (0.53–0.78)	0.68 (0.54–0.82)	0.78 (0.67–0.9)
rs1801131	G	Rectal 3-year survival	13.73	30.23	0.3 (0.1–0.6)	Diff	0.0025	0.62 (0.54–0.69)	0.77 (0.7–0.84)	0.81 (0.75–0.88)
		Rectal Capecitabine—5-year relapse	53.33	30.56	3.4 (1.2–9.9)	Same	0.022	0.61 (0.51–0.7)	0.7 (0.59–0.81)	0.79 (0.7–0.88)
		Rectal No chemotherapy—5-year relapse	54.05	20	13.1 (2.5–115)	Same	0.0065	0.67 (0.56–0.78)	0.75 (0.63–0.88)	0.9 (0.83–0.98)
rs1801159	C	III+IV 5-year relapse	7.69	22.33	0.3 (0.1–0.9)	-	0.0479	0.62 (0.54–0.71)	0.71 (0.6–0.82)	0.76 (0.66–0.87)
rs1801265	G	Right 5-fluorouracil—5-year relapse	15.38	48	0 (0–0)	-	0.0423	0.66 (0.52–0.8)	0.86 (0.74–0.99)	1 (1–1)
		Left No chemotherapy—5-year relapse	13.33	4.63	4 (1–16)	-	0.0443	0.64 (0.48–0.79)	0.7 (0.55–0.84)	0.81 (0.69–0.94)
		Left No chemotherapy—5-year relapse	34.21	16.05	3.7 (1.3–11)	-	0.0174	0.62 (0.51–0.72)	0.71 (0.6–0.81)	0.77 (0.67–0.87)
rs1045642	A	III+IV Capecitabine—5-year relapse	44.05	65.85	0.3 (0.1–0.9)	Same	0.0261	0.6 (0.52–0.68)	0.76 (0.67–0.84)	0.81 (0.73–0.88)
rs1128503	A	I+II 5-fluorouracil—5-year relapse	38.89	17.86	3.5 (1.1–13)	Diff	0.0376	0.6 (0.51–0.68)	0.63 (0.52–0.75)	0.71 (0.6–0.81)

**Table 3:** Suggestive (*p* < 5 × 10^−6^) SNPs in the analyzed outcomes. A1, tested allele; A2, other allele; OR (95% CI), odds ratio, and 95% confidence interval; *p*, the *p*-value of the additive association analysis; Freq, frequency of A1 allele in our cohort; Freq EUR, frequency of A1 allele in 1 KG European cohort.

Leading SNP	Position	Gene	A1	A2	Outcome	OR (95%CI)	P	Freq	Freq EUR
rs11207633	1:61007182	LINC01748	G	A	I+II 1-year survival	6.2 (2.8–13.4)	4.5 × 10^−6^	0.36	0.33
rs6659829	1:89477830	GBP3	C	T	No chemotherapy—5-year relapse	5.3 (2.6–10.4)	2 × 10^−6^	0.13	0.17
					I+II No chemotherapy—5-year relapse	5.7 (2.7–11-9)	4.8 × 10^−6^		
rs12477805	2:241016702	Upstream of NDUFA10	T	C	3-year survival	2.2 (1.6–3.1)	1.3 × 10^−6^	0.32	0.34
rs75254405	2:43852198	Upstream of THADA and PLEKHH2	C	T	I+II 5-year survival	6.1 (2.8–13.2)	3.8 × 10^−6^	0.06	0.06
rs17821546	2:108926033	SULT1C2	G	A	I+II 3-year survival	21.9 (6.1–78.6)	2.2 × 10^−6^	0.03	0.04
rs6736446	2:170967324	Downstream of UBR3, upstream of MY03B	A	G	Left 3-year survival	6.1 (2.8–13.1)	3.2 × 10^−6^	0.21	0.24
rs62240726	3:12930641	Downstream of IQSEC1	G	A	1-year survival	21.4 (5.9–78)	3.5 × 10^−6^	0.01	0.02
rs4688169	3:63439414	SYNPR, SYNPR-AS1	A	G	5-year survival	7.7 (3.2–18.6)	4.9 × 10^−6^	0.03	0.02
rs61471537	3:78102343	-	A	G	Rectal chemotherapy	0.2 (0.1–0.4)	3.9 × 10^−6^	0.20	0.31
rs1347485	4:30413696	-	G	A	3-year survival	8.3 (3.5–19.7)	1.6 × 10^−6^	0.02	0.04
					5-year survival	7.3 (3.1–17.1)	4.6 × 10^−6^		
					I+II 3-year survival	16.8 (5.3–53.9)	1.9 × 10^−6^		
rs852602	5:10898799	Downstream of CTNND2	T	A	Left 5-year survival	0.2 (0.1–0.4)	4.9 × 10^−6^	0.44	0.45
rs268718	5:33353086	Upstream of TARS1	A	G	I+II Capecitabine—5-year relapse	7.9 (3.3–18.9)	2.8 × 10^−6^	0.19	0.15
rs10941315	5:36732788	Downstream of SLCC1A3	T	G	Chemotherapy	2.3 (1.6–3.4)	3.2 × 10^−6^	0.40	0.48
rs6889868	5:144117510	-	T	C	3-year survival	3.2 (2–5)	6.4 × 10^−7^	0.13	0.14
rs10515827	5:160754957	GABRB2	T	C	I+II chemotherapy	6.3 (2.9–14)	4.9 × 10^−6^	0.12	0.17
rs4712605	6:21331584	Upstream of CDKAL1	A	G	1-year survival	4.9 (2.5–9.7)	2.9 × 10^−6^	0.05	0.06
					I+II 1-year survival	11.9 (4.1–34.1)	4.1 × 10^−6^		
rs1383747	6:113250149	-	A	G	5-year relapse	7.4 (3.2–17)	2.4 × 10^−6^	0.06	0.06
rs12193849	6:115771573	-	G	A	5-year relapse	5.9 (2.8–12.8)	3.6 × 10^−6^	0.07	0.09
rs10872669	6:151515172	Downstream of LOC102723831	A	G	1-year survival	4.2 (2.3–7.7)	2.1 × 10^−6^	0.08	0.11
rs11766180	7:67155516	-	T	C	5-year relapse	9.4 (3.6–24.5)	4.7 × 10^−6^	0.04	0.03
rs11761419	7:67185423	-	A	C	I+II 5-year relapse	41.3 (8.5–199)	3.6 × 10^−6^	0.05	0.03
rs75231954	7:90917290	Downstream of CDK14	A	G	I+II 5-year relapse	7.3 (3.1–17.1)	4.2 × 10^−6^	0.15	0.13
rs17831626	8:128080423	Upstream of PCAT2	T	G	5-year survival	0.5 (0.4–0.7)	3.6 × 10^−6^	0.45	0.42
					I+II 5-year survival	0.3 (0.2–0.5)	4.9 × 10^−6^		
rs11167104	8:142984200	-	T	C	I+II chemotherapy	0.3 (0.2–0.5)	4.6 × 10^−6^	0.42	0.47
rs72689069	8:143647614	Downstream of ADGRB1	T	C	Rectal 1-year survival	20.6 (5.7–74.3)	3.9 × 10^−6^	0.05	0.02
rs72768282	10:4829609	Upstream AKR1E2	C	A	Rectal 1-year survival	14.4 (4.8–43)	1.7 × 10^−6^	0.07	0.07
rs7074392	10:8518707	-	A	G	1-year survival	2.9 (1.8–4.7)	3.7 × 10^−6^	0.34	0.37
rs12267628	10:13282397	Upstream of UCMA	A	T	Rectal 1-year survival	21.9 (6.1–78.9)	2.3 × 10^−6^	0.04	0.09
rs10845123	12:10523900	KLRK1-AS1	A	G	5-year survival	2.9 (2–4.2)	9.6 × 10^−9^	0.24	0.26
rs4586220	12:22089348	ABCC9	G	A	1-year survival	6.9 (3.1–15.6)	3.4 × 10^−6^	0.03	0.03
rs7980214	12:31113401	TSPAN11	C	T	5-year survival	2.1 (1.5–2.8)	2.4 × 10^−6^	0.42	0.44
rs11051189	12:31122474	TSPAN11	C	T	1-year survival	2.9 (1.8–4.5)	2 × 10^−6^	0.29	0.31
rs7298118	12:111837285	Upstream of SH2B3	G	A	III+IV 3-year survival	3.4 (2.1–5.8)	2.5 × 10^−6^	0.3	0.22
rs9788099	12:132415557	PUS1	A	G	3-year survival	3.3 (1.9–5.4)	3.1 × 10^−6^	0.10	0.11
					Rectal 3-year survival	9.1 (3.6–22.9)	2.6 × 10^−6^		
rs61972489	13:100085274	Downstream of UBAC2	A	G	Rectal 3-year survival	7.4 (3.3–16.8)	1.4 × 10^−6^	0.09	0.07
rs9586086	13:103881964	-	A	G	3-year survival	2.2 (1.6–3)	1.8 × 10^−6^	0.42	0.31
rs72669827	14:33198189	AKAP6	A	G	chemotherapy	9.8 (3.9–25)	1.6 × 10^−6^	0.02	0.02
rs74622080	14:92762276	Upstream SLC24A4	T	G	III+IV 5-year survival	3.9 (2.2–7.2)	4.7 × 10^−6^	0.16	0.11
rs61991339	14:93867368	UNC79	C	T	1-year survival	4.2 (2.4–7.3)	7.7 × 10^−7^	0.14	0.17
rs13338718	16:26173425	Downstream of HS3ST4	T	C	Rectal 1-year survival	8.5 (3.5–20.3)	1.8 × 10^−6^	0.11	0.05
rs117046148	17:77264440	RBFOX3	A	G	I+II 3-year survival	17.5 (5.3–57.9)	2.5 × 10^−6^	0.02	0.04
rs490065	18:8075154	PTPRM	A	G	III+IV 5-year survival	0.3 (0.2–0.5)	3.6 × 10^−6^	0.37	0.37
rs12455842	18:33842286	MOCOS	C	T	Capecitabine—5-year relapse	4.5 (2.4–8.6)	4.3 × 10^−6^	0.14	0.8
rs34945948	19:41340842	Downstream of CYP2A6	G	A	I+II 5-year survival	4.3 (2.3–8.2)	4.6 × 10^−6^	0.12	0.16
rs141950185	19:51251682	Upstream of SHANK1, GPR32	G	T	I+II No chemotherapy—5-year relapse	9.4 (3.6–24.6)	4.7 × 10^−6^	0.07	0.08
rs6132492	20:22193192	-	A	G	1-year survival	2.6 (1.8–3.9)	1.3 × 10^−6^	0.40	0.49
rs6088387	20:32629322	RALY	T	G	Chemotherapy	6.5 (3.1–13.7)	7.9 × 10^−7^	0.06	0.07
rs371484	20:42359483	Upstream of GTSF1L	G	A	I+II 3-year survival	11.3 (4.1–31.3)	3.4 × 10^−6^	0.05	0.06
rs68035978	21:28101359	-	C	T	No chemotherapy—5-year relapse	0.2 (0.1–0.4)	4.3 × 10^−6^	0.22	0.28

## Data Availability

The data presented in this study are available on request from the authors. The data are not publicly available due to ethical reasons (genotype data cannot be shared).

## Supplementary Materials

The following supporting information can be downloaded at: https://www.mdpi.com/article/10.3390/cancers15194688/s1, Supplementary Table S1: SNPs previously associated with CRC outcomes analyzed in this work.
