# Supplementary material for: Genetic Variants as Predictors of the Success of Colorectal Cancer Treatments
Source: Cancers (Basel). 2023 Sep 22;15(19):4688. doi: 10.3390/cancers15194688 (PMC10571592; doi:10.3390/cancers15194688)
Supplement: Supplementary file 1 [file cancers-15-04688-s001.zip › cancers-2578827-supplementary.pdf]

Genetic Variants as Predictors of the Success of Colorectal Cancer Treatments

Koldo Garcia-Etxebarria, Ane Etxart, Maialen Barrero, Beatriz Nafria, Nerea Miren Segues Merino, Irati Romero-Garmendia, Ajay Goel, Andre Franke, Mauro D’Amato, Luis Bujanda

Item	Page
**Supplementary Table S1**: SNPs previously associated with CRC outcomes analysed in this work.	2

Supplementary Table S1: SNPs previously associated with CRC outcomes analysed in this work.

Outcome	SNP	Position	Effect Allele	Effect	Reference
Metastatic colorectal cancer survival in treatment with chemotherapy plus biologics	rs898838	2:18039508	G	HR=0.45	[1]
	rs16985284	2:18735164	C	HR=0.12	
	rs2461035	8:80590137	G	HR=0.72	
	rs11246159	11:436461	G	HR=0.17	
	rs7300446	12:16674220	G	HR=1.56	
	rs11644916	16:359567	A	HR=1.4	
Survival in rectal cancer	rs16867335	2:181458934	T	HR=3.27	[2]
	rs6854845	4:75746665	T	HR=4.12	
	rs17026425	4:150672514	A	HR=5.06	
	rs157411	5:67294907	G	HR=2.89	
	rs17057166	5:159248014	T	HR=5.56	
	rs4868304	5:173131457	T	HR=2.91	
	rs4959799	6:3295028	C	HR=3.5	
	rs1573948	6:6786425	C	HR=2.71	
	rs10275272	7:19160897	T	HR=3.19	
	rs3781663	11:69999252	G	HR=2.24	
	rs7004484	8:132934538	C	HR=2.79	
	rs11138220	9:82060528	G	HR=2.76	
	rs1555895	10:837407	A	HR=2.94	
	rs1570271	10:115288501	A	HR=3.66	
	rs9419702	10:133531153	C	HR=2.54	
	rs10152207	15:38129779	A	HR=3.06	
	rs338389	15:68260008	G	HR=3.4	
Survival in microsatellite instability low/stable colorectal cancer	rs6720296	2:45408269	C	HR=1.74	
	rs17048372	2:115213756	T	HR=1.91	
	rs10040610	5:6353815	C	HR=1.96	
	rs13180087	5:45265768	-	HR=2.06	
	rs1493383	5:152991398	T	HR=1,81	
	rs12187751	5:162585645	G	HR=2.48	
	rs17087282	6:156588020	A	HR=2.47	
	rs1998584	9:13704539	T	HR=1.76	
	rs1407508	9:101644538	C	HR=2.53	
	rs912294	13:31698294	A	HR=1.67	
Progression free survival in metastatic colorectal cancer (CAPOX-B vs CAPOX-B plus cetuximab)	rs4377367	2:131724905	C	HR=1.59	[3]
	rs2073016	6:41020922	T	HR=1.62	
	rs2936519	8:6639240	A	HR=1.89	
Progression free survival in metastatic colorectal cancer (chemotherapy interaction)	rs885036	2:99304794	A	HR=2.13	
Survival in colorectal cancer	rs12224794	11:41068900	-	HR=1.15	[4]
Survival in colorectal cancer (non-distant metastatic)	rs1075232	15:31741216	A	HR=1.49	
Survival in colorectal cancer (distant metastatic)	rs209489	6:53177678	C	HR=1.77	
	rs1442089	18:51107079	C	HR=1.83	
	rs1372474	18:51116832	G	HR=1.53	
FOLFIRI success	rs1054190	3:119536718	T	HR=0.65	[5]
	rs7910212	10:6009126	C	HR=1.55	
	rs7299460	12:48296268	C	HR=0.22	
	rs7179840	15:67458930	C	HR=0.65	
Bevacizumab success	rs16852804	2:215672527	A	OR=1.34	[6]
	rs3795897	2:236424382	A	OR=1.97	
	rs680949	9:79298133	G	OR=1.57	
5-fluoracile toxicity	rs1801131	1:11854476	C	Higher risk	[7]
	rs1801133	1:11856378	T	Higher risk	[8]
	rs55886062	1:97981343	G	RR=2.81	[9]
	rs1801160	1:97770920	A	OR=1.7	[10]
	rs1801159	1:97981395	-	-	[11]
	rs1801265	1:98348885	C	-	[12]
	rs2297595	1:98165091	G	OR=5.94	[13]
5-fluoracile and capecitabine toxicity	rs1045642	7:87138645	C	Higher risk	[14,15]
Capecitabine toxicity	rs1128503	7:87179601	C	Higher risk	
	rs2032582	7:87160618	G	Higher risk	

rs2072671	1:20915701	A	Higher risk	[15]
rs602950	1:20915531	C	Higher risk	[16]
rs532545	1:20915172	T	Higher risk	
rs3918290	1:97915614	A	OR=12	[17]

---

1. Innocenti, F.; Sibley, A.B.; Patil, S.A.; Etheridge, A.S.; Jiang, C.; Ou, F.-S.; Howell, S.D.; Plummer, S.J.; Casey, G.; Bertagnolli, M.M.; et al. Genomic Analysis of Germline Variation Associated with Survival of Patients with Colorectal Cancer Treated with Chemotherapy Plus Biologics in CALGB/SWOG 80405 (Alliance). *Clin Cancer Res* **2021**, *27*, 267–275, doi:10.1158/1078-0432.CCR-20-2021.

2. Xu, W.; Xu, J.; Shestopaloff, K.; Dicks, E.; Green, J.; Parfrey, P.; Green, R.; Savas, S. A Genome Wide Association Study on Newfoundland Colorectal Cancer Patients' Survival Outcomes. *Biomark Res* **2015**, *3*, 6, doi:10.1186/s40364-015-0031-6.

3. Pander, J.; van Huis-Tanja, L.; Böhringer, S.; van der Straaten, T.; Gelderblom, H.; Punt, C.; Guchelaar, H.-J. Genome Wide Association Study for Predictors of Progression Free Survival in Patients on Capecitabine, Oxaliplatin, Bevacizumab and Cetuximab in First-Line Therapy of Metastatic Colorectal Cancer. *PLoS One* **2015**, *10*, e0131091, doi:10.1371/journal.pone.0131091.

4. Phipps, A.I.; Passarelli, M.N.; Chan, A.T.; Harrison, T.A.; Jeon, J.; Hutter, C.M.; Berndt, S.I.; Brenner, H.; Caan, B.J.; Campbell, P.T.; et al. Common Genetic Variation and Survival after Colorectal Cancer Diagnosis: A Genome-Wide Analysis. *Carcinogenesis* **2016**, *37*, 87–95, doi:10.1093/carcin/bgv161.

5. De Mattia, E.; Polesel, J.; Roncato, R.; Labriet, A.; Bignucolo, A.; Gagno, S.; Buonadonna, A.; D'Andrea, M.; Lévesque, E.; Jonker, D.; et al. IL15RA and SMAD3 Genetic Variants Predict Overall Survival in Metastatic Colorectal Cancer Patients Treated with FOLFIRI Therapy: A New Paradigm. *Cancers* **2021**, *13*, doi:10.3390/cancers13071705.

6. Quintanilha, J.C.F.; Wang, J.; Sibley, A.B.; Xu, W.; Espin-Garcia, O.; Jiang, C.; Etheridge, A.S.; Ratain, M.J.; Lenz, H.-J.; Bertagnolli, M.; et al. Genome-Wide Association Studies of Survival in 1520 Cancer Patients Treated with Bevacizumab-Containing Regimens. *Int J Cancer* **2022**, *150*, 279–289, doi:10.1002/ijc.33810.

7. Thomas, F.; Motsinger-Reif, A.A.; Hoskins, J.M.; Dvorak, A.; Roy, S.; Alyasiri, A.; Myerson, R.J.; Fleshman, J.W.; Tan, B.R.; McLeod, H.L. Methylenetetrahydrofolate Reductase Genetic Polymorphisms and Toxicity to 5-FU-Based Chemoradiation in Rectal Cancer. *British Journal of Cancer* **2011**, *105*, 1654–1662, doi:10.1038/bjc.2011.442.

8. Derwinger, K.; Wettergren, Y.; Odin, E.; Carlsson, G.; Gustavsson, B. A Study of the MTHFR Gene Polymorphism C677T in Colorectal Cancer. *Clin Colorectal Cancer* **2009**, *8*, 43–48, doi:10.3816/CCC.2009.n.007.

9. Meulendijks, D.; Henricks, L.M.; Sonke, G.S.; Deenen, M.J.; Froehlich, T.K.; Amstutz, U.; Largiadèr, C.R.; Jennings, B.A.; Marinaki, A.M.; Sanderson, J.D.; et al. Clinical Relevance of DPYD Variants c.1679T>G, c.1236G>A/HapB3, and c.1601G>A as Predictors of Severe Fluoropyrimidine-Associated Toxicity: A Systematic Review and Meta-Analysis of Individual

Patient Data. *Lancet Oncol* **2015**, *16*, 1639–1650, doi:10.1016/S1470-2045(15)00286-7.
10. Boige, V.; Vincent, M.; Alexandre, P.; Tejpar, S.; Landolfi, S.; Le Malicot, K.; Greil, R.; Cuyle, P.J.; Yilmaz, M.; Faroux, R.; et al. DPYD Genotyping to Predict Adverse Events Following Treatment With Fluorouracil-Based Adjuvant Chemotherapy in Patients With Stage III Colon Cancer: A Secondary Analysis of the PETACC-8 Randomized Clinical Trial. *JAMA Oncol* **2016**, *2*, 655–662, doi:10.1001/jamaoncol.2015.5392.
11. Zhang, H.; Li, Y.-M.; Zhang, H.; Jin, X. DPYD*5 Gene Mutation Contributes to the Reduced DPYD Enzyme Activity and Chemotherapeutic Toxicity of 5-FU. *Medical Oncology* **2007**, *24*, 251–258, doi:10.1007/BF02698048.
12. Baskin, Y.; Amirfallah, A.; Unal, O.U.; Calibasi, G.; Oztop, I. Dihydropyrimidine Dehydrogenase 85T>C Mutation Is Associated With Ocular Toxicity of 5-Fluorouracil: A Case Report. *American Journal of Therapeutics* **2015**, *22*.
13. Falvella, F.S.; Cheli, S.; Martinetti, A.; Mazzali, C.; Iacovelli, R.; Maggi, C.; Gariboldi, M.; Pierotti, M.A.; Di Bartolomeo, M.; Sottotetti, E.; et al. DPD and UGT1A1 Deficiency in Colorectal Cancer Patients Receiving Triplet Chemotherapy with Fluoropyrimidines, Oxaliplatin and Irinotecan. *Br J Clin Pharmacol* **2015**, *80*, 581–588, doi:10.1111/bcp.12631.
14. Gonzalez-Haba, E.; García, M.I.; Cortejoso, L.; López-Lillo, C.; Barrueco, N.; García-Alfonso, P.; Alvarez, S.; Jiménez, J.L.; Martín, M.L.; Muñóz-Fernández, M.A.; et al. ABCB1 Gene Polymorphisms Are Associated with Adverse Reactions in Fluoropyrimidine-Treated Colorectal Cancer Patients. *Pharmacogenomics* **2010**, *11*, 1715–1723, doi:10.2217/pgs.10.159.
15. García-González, X.; Cortejoso, L.; García, M.I.; García-Alfonso, P.; Robles, L.; Grávalos, C.; González-Haba, E.; Marta, P.; Sanjurjo, M.; López-Fernández, L.A. Variants in CDA and ABCB1 Are Predictors of Capecitabine-Related Adverse Reactions in Colorectal Cancer. *Oncotarget* **2015**, *6*, 6422–6430, doi:10.18632/oncotarget.3289.
16. Loganayagam, A.; Arenas Hernandez, M.; Corrigan, A.; Fairbanks, L.; Lewis, C.M.; Harper, P.; Maisey, N.; Ross, P.; Sanderson, J.D.; Marinaki, A.M. Pharmacogenetic Variants in the DPYD, TYMS, CDA and MTHFR Genes Are Clinically Significant Predictors of Fluoropyrimidine Toxicity. *British Journal of Cancer* **2013**, *108*, 2505–2515, doi:10.1038/bjc.2013.262.
17. Deenen, M.J.; Tol, J.; Burylo, A.M.; Doodeman, V.D.; de Boer, A.; Vincent, A.; Guchelaar, H.-J.; Smits, P.H.M.; Beijnen, J.H.; Punt, C.J.A.; et al. Relationship between Single Nucleotide Polymorphisms and Haplotypes in DPYD and Toxicity and Efficacy of Capecitabine in Advanced Colorectal Cancer. *Clin Cancer Res* **2011**, *17*, 3455–3468, doi:10.1158/1078-0432.CCR-10-2209.
